# Studies on the Application of PGM Nanocatalysts from Spent Automotive Converters for Degradation of Ibuprofen in Aqueous Solutions

**DOI:** 10.3390/ijms25063147

**Published:** 2024-03-09

**Authors:** Zuzanna Wolańczyk, Wiktoria Stachowicz, Martyna Rzelewska-Piekut, Joanna Zembrzuska, Magdalena Regel-Rosocka

**Affiliations:** 1 Institute of Chemical Technology and Engineering, Faculty of Chemical Technology, Poznan University of Technology, ul. Berdychowo 4, 60-965 Poznan, Poland; zuzanna_wiecka@wp.pl (Z.W.); wiktoria.stachowicz@doctorate.put.poznan.pl (W.S.); martyna.rzelewska-piekut@put.poznan.pl (M.R.-P.); 2 Institute of Chemistry and Technical Electrochemistry, Faculty of Chemical Technology, Poznan University of Technology, ul. Berdychowo 4, 60-965 Poznan, Poland; joanna.zembrzuska@put.poznan.pl

**Keywords:** platinum group metals, ibuprofen degradation, nanoparticles, spent automotive converters

## Abstract

There is an increasing concern about the presence of various types of pharmaceuticals in drinking water, as long-term exposure of people to even low concentrations of drugs can lead to many problems, such as endocrine disorders or drug resistance. As the removal in sewage treatment plants is not effective enough, as indicated, among others, by the EC and OECD reports, it is justified to search for new materials that will allow for an effective and rapid reduction of these pollutants in water. Therefore, in our work, catalytically active nanomaterials containing platinum group metals (PGMs) were synthesized from model and real multicomponent solutions and examined in reactions of organic compounds. The nanoparticles (NPs) were obtained from real solutions from the hydrometallurgical processing of spent automotive converters (SACs), and to the best of our knowledge, the novelty of the proposed paper is the application of solutions from SAC processing as precursors for PGM–NPs. The synthesized PGM–NPs were deposited on a support (TiO_2_), characterized and, finally, examined as nanocatalysts in a degradation reaction of ibuprofen (IB) from model aqueous solutions. The degree of IB degradation reached more than 90%. The main products of IB degradation were p-isobutylphenol and CO_2_.

## 1. Introduction

The pharmaceutical industry is one of the fastest growing industries. This is influenced by a number of factors, including an aging population, an increase in the incidence of chronic and civilization-related diseases, and increasing funding for research and development. The increasing consumption of pharmaceuticals year after year contributes to an increase in the scale of production and, consequently, also an increase in the amount of pharmaceuticals released into the environment. The main sources of pollution by these chemical compounds are primarily pharmaceutical companies, as well as human and animal health care facilities (hospitals, medical, dental and veterinary clinics, clinics, sanatoriums, diagnostic laboratories, etc.) [1,2,3,4]. A significant amount of pharmaceuticals and their metabolites also enter the environment from households and animal farms [5]. This is due to the excessive use of medicinal substances that are not completely absorbed by the body, and the fact that most wastewater treatment plants are unable to completely remove pharmaceuticals and their metabolites from discharged wastewater [2]. Part of the drug taken (up to 90%) is excreted from the human and animal body and enters sewage or soil, and then groundwater [6].

A pressing environmental concern is the presence of pharmaceuticals in waters, known for their potential harmful impact on human and animal health. The most common drugs that appear in water and soil include non-steroidal anti-inflammatory drugs (NSAIDs) and analgesics, antibiotics, hormonal agents, *β*-blockers, lipid regulators, psychotropic and antiepileptic drugs, and other active substances (including caffeine) [2,4,5,7]. The pharmaceutical industry’s dynamic growth and the increasing use of pharmaceuticals require innovative approaches to address their entry into the ecosystem. Conventional sewage treatment plants have proven insufficient in removing these substances, prompting the search for more effective methods for treating pharmaceutical contaminants in water [2,4,8].

Monitoring pharmaceuticals in water is in line with the EU’s strategic approach to pharmaceuticals in the environment: the One Health European Action Plan to combat antimicrobial resistance, recognizing the interconnections between human health, animal health, and the environment [9]. The EU 2022 watch list includes, among others: clotrimazole, fluconazole, the insecticide and the veterinary medicinal product fipronil, and the antibiotics clindamycin and ofloxacin [10]. Use of PGMs in the form of new catalytically active nanomaterials aligns with the European Commission’s planned activities to enhance wastewater treatment efficiency of pharmaceuticals in the forthcoming years. In the degradation of pharmaceuticals, photodegradation reactions are facilitated by PGM–NPs deposited on supports, with TiO_2_ [11,12,13] and Al_2_O_3_ [14] being commonly employed. The application of PGM–NPs on TiO_2_ significantly enhances the photolytic activity of the semiconductor. Notably, PGM-NPs have a larger surface area compared to microparticles, resulting in more efficient solute adsorption. For instance, regardless of the pH conditions, the presence of Pd on TiO_2_ enhances the rate of paracetamol decomposition and mineralization by at least a twofold factor [12]. Additionally, magnetic PGM–NPs were proposed as catalysts for the degradation of pharmaceuticals. Examples include magnetic TiO_2_@ZnFe_2_O_4_/Pd with an enhanced photocatalytic activity for the degradation of diclofenac [15], or Pd@Fe_3_O_4_ for the degradation of ibuprofen in a hybrid sono-electrolytical system [16]. It was observed that the addition of PGM–NPs increased the degradation rate of the processed pharmaceuticals. Generally, processes employing metal NPs as catalysts represent a highly promising approach for wastewater treatment. Beyond the significantly enhanced catalytic activity attributed to the high surface-area-to-volume ratio of nano-sized catalysts compared to bulk catalysts, environmental nanotechnology emphasizes the safe design of nanomaterials with potential environmental benefits, embracing the principles of “green nanoscience” [17].

Green nanoscience imposes the reduction of environmental impacts and process safety through the avoidance of hazardous compounds in NP formation systems and the promotion of benign reagents, solvents, or hazardless and bio-based NP feedstocks [17]. In this context, the biomimetic and bioinspired synthesis of nanomaterials has gained increasing interest. Biomimetic approaches can mimic the functions of natural materials/structures or the biological processes organisms use to produce substances or materials [18]. Bioinspired materials replicate the structure, properties, or function of natural materials, e.g., light-harvesting photonic materials that mimic photosynthesis, nanocatalysts inspired by structures of enzymes, and hybrid assemblies that mimic the hierarchical architecture of nacre, antler, or bone. These bioinspired materials, with their unique properties, are recognized as future technologies, drawing collaborations between global companies and scientists to explore and develop novel technologies based on designs inspired by nature.

Thus, our studies focus on using PGM–NPs obtained as a result of the hydrometallurgical process for recovering PGMs from SACs. These nanoparticles play a pivotal role in a model reaction, specifically the reduction of 4-NPh to 4-APh, as well as in the degradation of IB from simulated aqueous solutions. This approach reflects a contemporary process for PGM recovery and reuse, aligning seamlessly with the principles of a circular economy.

## 2. Results and Discussion

### 2.1. Catalysts from Model Solutions

**Figure 1:**
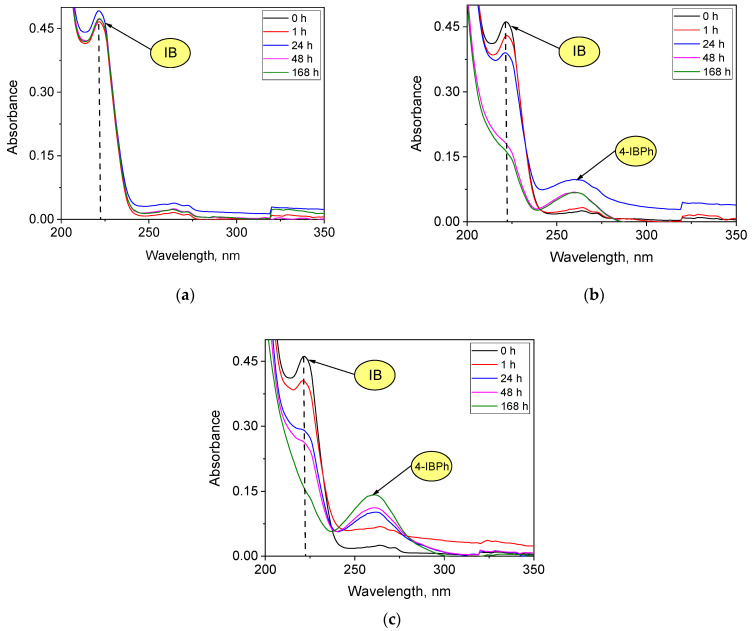
Change in IB spectrum during degradation in the absence of exposure to UV–Vis light: (**a**) without a catalyst, (**b**) with TiO_2_ alone, (**c**) with 1% Pd@TiO_2_ (300 rpm, max. 168 h, ambient temperature, amount of catalyst 150 mg, solution volume 150 cm^3^).

As shown in our previous studies [19,20], the use of a TiO_2_ support facilitated the straightforward separation of the obtained Pt–NPs from the reaction mixture. The synthesized materials underwent preliminary testing in a model catalytic reduction reaction of 4-NPh to 4-APh. This reaction proved the catalytic activity of the newly formed nanomaterials and showed reusability for a minimum of seven cycles when catalyzing the model reduction reaction of 4-NPh to 4-APh without a significant decline in catalytic activity. These positive results inspired us to investigate the effect of the PGMs@TiO_2_ presence on the degradation of ibuprofen (IB) without UV–Vis light exposure (Figure 1). An amount of 1% of Pd@TiO_2_ or Pt@TiO_2_ formed from model solutions was chosen as the nanocatalyst. These nanomaterials were characterized elsewhere (TEM, AFM images, and the distribution of particle size in Supplementary Materials of [20]), and it was confirmed using TEM images that the size of PGM–NPs was less than 10 nm.

**Scheme 1:**
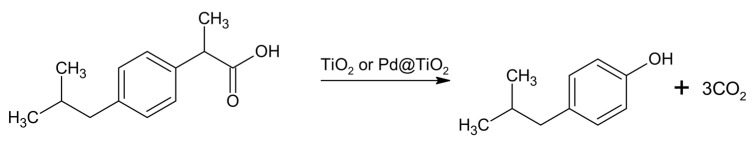
The reaction of IB degradation to 4-IBPh.

The degradation reaction of IB was conducted for 7 days in the absence of exposure to UV–Vis light. The extended reaction time was due to the lack of a visible change in the conversion degree of IB after several hours of the experiment. A characteristic wavelength maximum at 222 nm corresponding to the presence of IB is visible in Figure 1. However, during the course of the reaction, a new maximum appears at a wavelength of 260 nm and can be attributed to the formation of 4-isobutylphenol (4-IBPh). For the first 24 h of conducting the IB degradation, the presence of Pd–NPs in the catalyst seems to have a slight effect on the IB conversion compared to the reaction in the presence of TiO_2_ alone. Namely, in the presence of TiO_2_ or 1% Pd@TiO_2_, the conversion degree of IB was 12 and 8% after 1 h, respectively, while after 7 days (168 h) of the reaction, it reached 60 and 65%, respectively. However, after 24 h the IB absorbance at the maximum visibly decreases while 4-IBPh maximum increases (Figure 1b,c). Finally, after 168 h of the reaction, the maximum of the degradation product (4-IBPh) showed a significantly higher absorbance after the reaction was catalyzed by 1% Pd@TiO_2_ than by TiO_2_ alone (Figure 1b,c). It is evident that the presence of the nanocatalyst, or at the very least the TiO_2_ support alone, is necessary for the degradation reaction of IB to occur. In the absence of the catalyst, even after 7 days of degradation, no IB conversion was observed (Figure 1a). The degradation reaction of IB to 4-IBPh can be expressed as follows (Scheme 1):

The increase in pH (Table 1) after 7 days of reaction time indirectly confirms the formation of the second reaction product (Scheme 1), namely CO_2_. These initial investigations were carried out at a single pH value (4.7). As it was pointed out by previous research which suggested that at a pH close to the pK_a_, the interaction between IB and the catalyst is enhanced due to greater adsorption of the undissociated IB onto the catalyst surface [21]. At a pH near 4.9, the dissociation of the IB carboxylic acid group decreases, leading to the increased hydrophobicity of IB and improved surface contact with the catalyst. However, a more comprehensive examination of the influence of pH on the degree of IB conversion is required to validate this assumption further.

Nevertheless, the IB degradation is much more efficient while enhanced by UV–Vis light. Generally, the first step of the formation of intermediates in the photodegradation of IB has been attributed to a multistage reaction of the introduction of hydroxyl groups (–OH) into the isobutyl group, propionic acid, or benzene ring via a ^•^OH radical. Various degradation products have been reported such as (4-methylphenyl)propanoic acid, 4-(2-methylpropyl)acetophenone, 1-(4-isobutylphenyl)-1-ethanol, 4-(1-hydroxy-2-methylpropyl)acetophenone, 4-isobutyl-benzaldehyde, 4-ethylbenzaldehyde, 3-methyl-4-oxopentanoic acid, 4-hydroxycatechol, or 4-ethylphenol. Subsequently, products of the first step are further degraded in the second step reactions such as decarboxylation, demethylation, and hydroxylation [22,23]. Finally, a ring cleavage might occur and further mineralization to CO_2_ and H_2_O is possible [24]. Villavicencio et al. [22] and Tian et al. [25] identified 4-isobutylphenol as a result of IB photodegradation, for example, in the presence of a Ag/Ag_2_O nanocomposite [22]. The formation of 4-IBPh as the main product of IB degradation likely occurs due to the decarboxylation of the substrate resulting from its interactions with the three distinct radicals (^•^OH, O_2_^•−^, and ^•^OOH) generated in the reaction sequence, mediated by the catalysts used [22,23]. Among these radicals, the hydroxyl radical (^•^OH) exhibits a superior oxidation capacity and is responsible for an attack on the high electronic density region of IB molecule located close to the aromatic ring (C=C bond).

**Figure 2:**
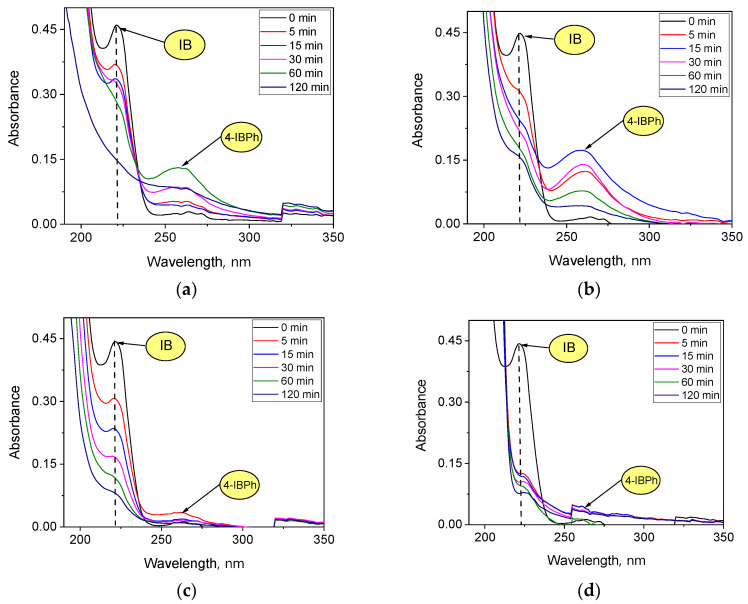
UV–Vis spectra of IB during the degradation enhanced by UV–Vis light (**a**) without a catalyst, (**b**) in the presence of TiO_2_, (**c**) Pd@TiO_2_, and (**d**) Pt@TiO_2_ during (300 rpm, 2 h, ambient temperature, catalyst mass 150 mg, solution volume 150 cm^3^).

Photoirradiation of the TiO_2_ support plays an important role in radical generation, resulting in the formation of an electron in the conduction band and a hole in the valence band. Due to the photoinduced separation of charges on the semiconductor surface, exchange reactions involving electrons take place at the interface between water and the semiconductor. The interaction of photo-generated electrons with adsorbed oxygen molecules leads to the formation of the superoxide radical anion O_2_^•−^. Additionally, TiO_2_ participates in ^•^OH generation through the valence band hole oxidation of adsorbed water or hydroxyl anions. Subsequently, the formed ^•^OH oxidizes the adsorbed pharmaceutical molecules. Alternatively, holes may directly oxidize the adsorbed organic compounds [26]. Thus, the impact of the exposure to UV–Vis light (for 2 h) on the degradation of IB was investigated in the presence of TiO_2_ and PGMs@TiO_2_ nanocatalysts (Figure 2).

**Figure 3:**
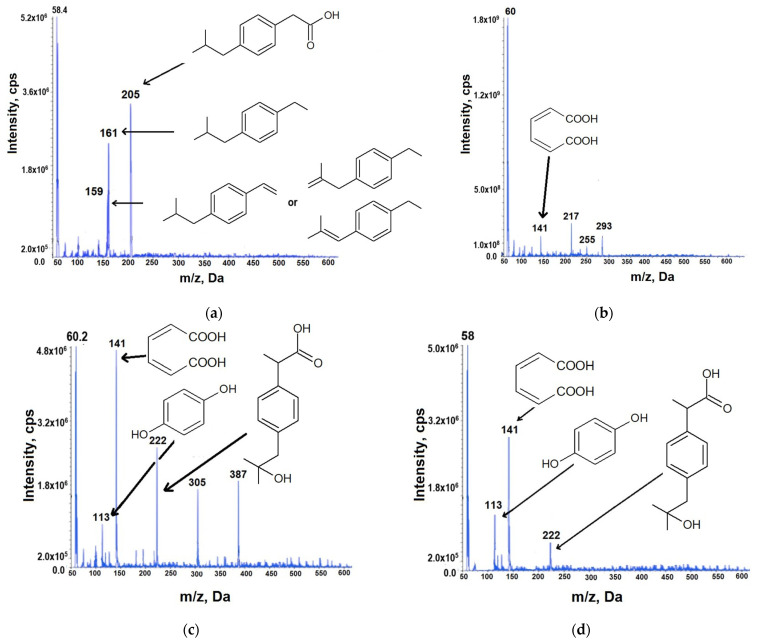
Negative ionization mass spectra of chromatographic peaks obtained (**a**) before reaction, after 120 min of the IB photodegradation reaction with exposure to UV–Vis light (**b**) after reaction without the catalyst, (**c**) after reaction in the presence of Pt@TiO_2_, (**d**) after reaction in the presence of Pd@TiO_2_.

In all spectra of the TiO_2_-mediated IB photodegradation, the disappearance of the maximum corresponding to the presence of IB (at 222 nm) is evident. In the spectra of the IB solutions after the reaction without a catalyst or with TiO_2_, a significant maximum is noted at 262 nm, appearing as early as 5 min during the course of the reaction. Similar to the reactions conducted without exposure to the light, this maximum corresponds to the formation of 4-IBPh in the solution. However, the IB degradation in the presence of Pd@TiO_2_ and Pt@TiO_2_ results in a minor maximum at 262 nm. Simultaneously, the maximum corresponding to IB (222 nm) gradually diminishes for Pd@TiO_2_, and rapidly for Pt@TiO_2_. This observation implies that IB is converted into products other than 4-IBPh, for example, such as these proposed in the LC–MS/MS mass spectra of chromatographic peaks obtained after 120 min of the reaction course in the presence of the Pt@TiO_2_ and Pd@TiO_2_ nanocatalysts (Figure 3). 

In the IB sample before the reaction (*m*/*z* = 205, negative ionization), ions of 1-ethyl-isobutylbenzene (*m*/*z* = 161), and 1-isobutyl-4-vinylbenzene, 1-ethyl-4-(2-methylprop-1-en-1-yl)benzene, and 1-ethyl-4-(2-methylprop-2-en-1-yl) benzene (*m*/*z* = 159), resulting from the IB decarboxylation, are identified. The appearance of these products is likely due to hydrolysis rather than IB degradation, since the initial IB solution was not exposed to sunlight or a UV–Vis lamp before the reaction [27]. The reaction catalyzed by PGMs@TiO_2_ leads to lower molecular weight products, such as muconic acid (*m*/*z* = 141) and hydroquinone (*m*/*z* = 113) [28]. In contrast, the photodegradation without the catalyst and with the Pt@TiO_2_ resulted in products with higher masses than IB. It indicates that the oxidation of organic compounds might have occurred, resulting in 2-[4-(2-hydroxy-2-methylpropyl)phenyl]propanoic acid (*m*/*z* = 222). However, the precise determination of the mechanism and identification of the main reactive factor responsible for photocatalytic degradation requires further research using quenchers (isopropyl alcohol, ethanol, Na_2_EDTA, and p-benzoquinone) [29].

### 2.2. Catalyst from a Real Solution

**Figure A1:**
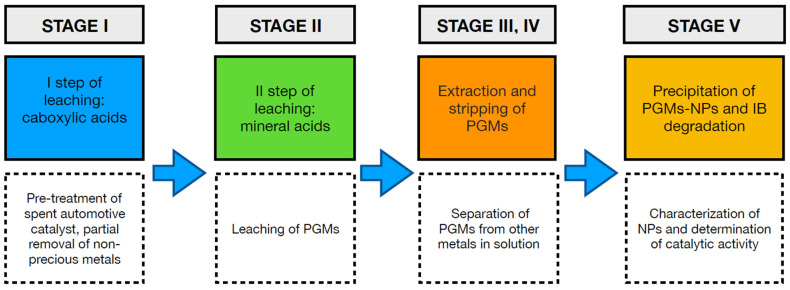
Scheme of the process for producing PGM-NPs from SACs.

The catalyst derived from a real solution was prepared in a four-stage process (see a scheme shown in Figure A1 in Appendix A), namely the SACs were leached with an oxalic acid solution, then with a HCl/H_2_O_2_/H_2_SO_4_ mixture. Subsequently, the PGMs were extracted from the leachate with Cyphos IL 101 at volume ratio of O/A = 6, and then stripped with 3 M HNO_3_ at A/O = 2 to enrich the stripping phase in Pt(IV) and Pd(II). Finally, NPs were formed from the stripping solution containing 89.4, 67.6, and 80.0 mg/dm^3^ of Pt(IV), Pd(II), and Fe ions, respectively. The total precipitation of NPs for the Pd, Pt, and Fe ions present in the real solution was determined with atomic absorption spectroscopy (AAS). The metal ion concentration was measured in the aqueous solutions before and after precipitation, and the mass balance indicated complete precipitation of the target metals. Additionally, our previous studies have demonstrated that the catalytic material PGMs@TiO_2_ is reusable for at least 7 cycles in a model reaction, reducing 4-NPh to 4-APh, without a decrease in catalytic activity [20].

**Figure A2:**
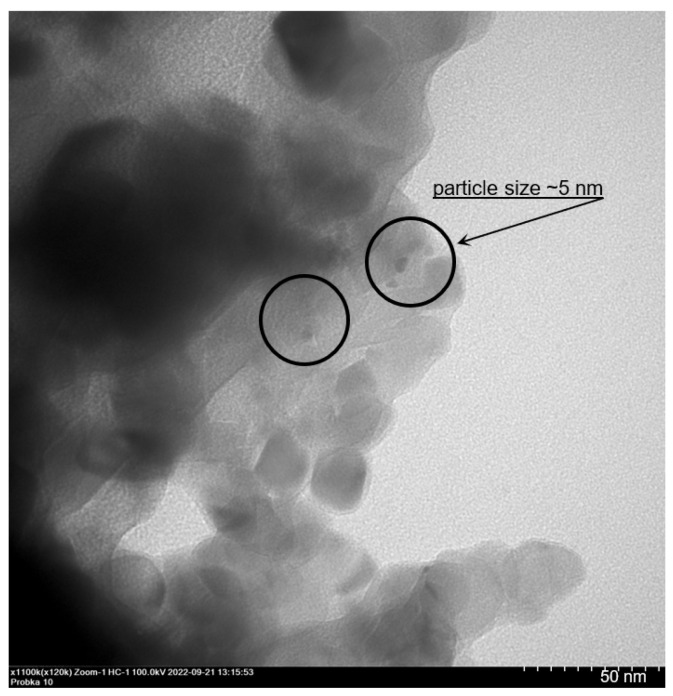
TEM image of Pt/Fe/Pd@TiO_2_ nanoparticles obtained from the real solution.

The TEM image of the PGM–NPs formed in the hydrometallurgical process distributed on a TiO_2_ support (Pt/Fe/Pd@TiO_2_) is shown in Figure A2 in Appendix A. The TEM, AFM images, and distribution of particle size for the NPs precipitated from real solutions are shown in our previous work [20]. The PGM–NPs were well dispersed on the TiO_2_ surface, and the particle size of the PGM–NPs obtained did not exceed 5 nm. Since the catalytic activity of NPs depends on their size, it is important to form an optimal particle size [30,31]. The smaller the NPs, the greater the number of catalytic centers available for the reactants. The optimal size varies depending on the reaction investigated, for example, for oxygen reduction in a fuel cell, the optimal Pt–NP size is suggested to be around 3 nm, whereas for methanol oxidation it is approximately 2 nm. Moreover, the catalysis of D-glucose oxidation confirmed that the highest catalytic activity was achieved for the smallest Pd–NPs supported on hypercrosslinked polystyrene (HPS) allowing for the optimal hydrophobicity–hydrophilicity balance with HPS [30]. The use of Pt/Fe/Pd@TiO_2_ nanoparticles obtained from the real solution for IB degradation was analyzed with UV–Vis spectroscopy and LC-MS/MS, and the results of the degree of IB conversion in the presence of catalysts precipitated from model and real solutions are compared in Table 2.

The challenges in acquiring spectra arose from difficulties in separating the Pt/Fe/Pd@TiO_2_ particles from the reaction mixture, hampering the determination of IB conversion via the UV–Vis technique. Consequently, there is no corresponding data in Table 2 for this technique.

The results presented in Table 2 indicate that the most effective IB degradation occurs in the presence of Pt@TiO_2_. The shift in pH of the IB solution from acidic conditions (4.7) to neutral (6.3–7.1) likely stems from the degradation of the carboxyl group of IB and the formation of CO_2_. Spectrophotometric analysis of the IB concentration changes indicates that IB degradation takes place even without a catalyst, but is less effective than in the presence of any of the produced catalysts. After 120 min of degradation, IB was converted by approximately 60%, and the pH slightly increased to 5. The use of Pt or Pd@TiO_2_ catalysts significantly enhanced the IB conversion by at least 20 percentage points. To confirm the catalytic activity of the obtained NPs and to verify the results from the UV–Vis spectra, the IB concentration in reaction samples was also determined using the LC–MS/MS technique (Table 2). Nevertheless, the efficacy of the LC–MS/MS technique when interacting with NPs becomes a potential challenge if proper separation is not achieved. This is primarily attributed to the nanometric dimensions of the particles within the analyzed solutions. Such a circumstance can adversely impact the cost-effectiveness of the analysis, as the presence of NPs may accelerate the wear of the chromatographic column or lead to capillary clogging.

The IB conversion values estimated from the LC–MS/MS results differ significantly from those obtained by UV–Vis spectroscopy. The values of the degree of IB conversion after 120 min of degradation, determined by LC–MS/MS, are high regardless of the presence of the catalyst and its type. The greatest differences in the degree of IB conversion were seen after 15 min of degradation. The IB degradation after 15 min of the reaction without catalyst was almost 40 percentage points lower than in the presence of Pt/Fe/Pd@TiO_2_. This may indicate that the use of Pt/Fe/Pd@TiO_2_ catalyst accelerates the IB degradation reaction at the initial stage of the reaction. Based on the IB degradation results obtained through LC–MS/MS analysis in the presence of PGM@TiO_2_ from both model and real solutions (Table 3), the kinetics of the reactions were examined. Assuming the IB degradation reaction follows first-order kinetics, the rate constants (k) at ambient temperature were determined using the following expression:

(1) $$ln\left(\frac{{IB}_{t}}{{IB}_{0}}\right)=-k\cdot t$$

where k represents the reaction rate constant, t denotes time, and the subscripts 0 and t stand for initial IB concentration, and IB concentration at the specified time, respectively.

Over the initial 30 min, the IB concentration gradually changed, while after more than half an hour, there was no significant change in the degree of IB conversion (Table 2). Consequently, the rate constant values were estimated within the initial 30 min period and are presented as averaged values for each catalyst employed (Table 3).

In the present investigation, the degradation of IB was observed to occur more rapidly in the presence of Pt@TiO_2_ compared to Pd@TiO_2_, with rate constants of 0.0877 min^−1^ and 0.0776 min^−1^, respectively. Remarkably, the rate constant shown by Pt@TiO_2_ was found to be tenfold greater than that reported for IB degradation in the presence of iron-doped mesoporous silica (Fe–FSM–16) [32]. This enhancement in catalytic activity highlights the performance of Pt@TiO_2_ in facilitating IB degradation, making it a promising material for pharmaceutical removal applications.

As expected, the degradation reaction proceeded at a considerably slower rate in the absence of PGM NPs on TiO_2_. However, the catalyst obtained from SAC hydrometallurgical treatment (Pt/Fe/Pd@TiO_2_) appeared to increase the rate constant of IB degradation, reaching 0.2205 min^−1^, in contrast to other materials synthesized (<0.09 min^−1^). The inclusion of Fe in Pt/Fe/Pd@TiO_2_ seems to play a crucial role in improving the catalytic properties of the resulting material. The specific mechanisms underlying the synergistic effects of Pt, Fe, and Pd in promoting IB degradation should be examined in detail in future investigations.

Fe–NPs are known to indirectly enhance catalysis by participating in Fenton-like reactions, wherein iron reacts with hydrogen peroxide (H_2_O_2_) to generate highly reactive hydroxyl radicals (^•^OH) [33,34]. Although H_2_O_2_ was not intentionally added during the course of the IB degradation in the presented studies, it is noteworthy that H_2_O_2_ can be formed in systems catalyzed with Fe–FSM–16, particularly under UV-induced photodegradation conditions [32]. In addition, the interaction between metal NPs and IB plays an important role in facilitating IB adsorption onto the catalyst surface, improving the contact between the catalyst and the target molecule, and thus promoting its degradation [35,36].

Summarizing, the use of PGM–NPs, particularly Pt@TiO_2_ and Pt/Fe/Pd@TiO_2_ catalysts, seems to be promising for enhancing the efficiency of IB degradation in aqueous solutions. The remarkable catalytic activity shown by these materials in these preliminary studies, encourages us to further research and explain the mechanisms governing the catalytic degradation of IB in the presence of Pt/Fe/Pd@TiO_2_, and to optimize the design of PGM-based catalysts for enhanced performance in environmental applications.

To sum up, the IB conversion values obtained in the present studies (Table 2) are compared to the degradation reported in the presence of NPs composed of various metals (Table 4). Notably, a wide variety of catalytic properties is noted, beginning with biogenic Pt and Pd–NPs, which are not active in IB degradation, followed by Ag/Ag_2_O (30%) and ZnO (60%), and culminating in complete IB conversion within 5 min using the photocatalyst TiO_2_ NPs. It appears that the Pt/Fe/Pd@TiO_2_ catalyst synthesized in this work, derived from the hydrometallurgical treatment of SACs, reaches 88% IB conversion in 5 min and 92% in 60 min, comparable to other efficient nanocatalysts such as MgFe_2_O_4_ or magnetic Pd@Fe_3_O_4_.

## 3. Materials and Methods

### 3.1. Reagents and Solutions

One-component model solutions with a composition similar to real solutions were prepared by dissolving the required amounts of PtCl_4_ (96%), or PdCl_2_ (99.9%) (Sigma Aldrich, Schnelldorf, Germany) in 0.1 M HCl (pure p.a., Chempur, Piekary Śląskie, Poland). Polyvinylpyrrolidone (PVP, M_w_ ≈ 55,000, Sigma Aldrich, Schnelldorf, Germany) was used as the stabilizing agent. Sodium borohydride (NaBH_4_) (>98.0%, Sigma Aldrich, Schnelldorf, Germany) was used as the reducer in the study to precipitate PGM–NPs from model solutions.

The real solution used in the research as a feed solution was from the hydrometallurgical treatment of Pt–Pd–Rh spent automotive converters (SACs). The SAC was processed according to the scheme presented in Figure A1 (Appendix A). The real solution applied for NP formation was obtained from the stripping step with 3 M HNO_3_ (pure p.a., Chempur, Piekary Śląskie, Poland). The concentrations of metal ions in the feed solutions used for Pt/Fe/Pd@TiO_2_ precipitation were as follows (in mg/dm^3^): 89.4 (Pt(IV)), 67.6 (Pd(II)), 80.0 (Fe ions), 1.0 (Mg(II)), 4.3 (Zn(II)), and 0.9 (Cu(II)).

### 3.2. Synthesis of Nanoparticles

The stabilizing agent PVP was added to the PGM precursor solution and mixed for 10 min. Next, the reducer was added drop by drop, while the solution was still being mixed. The pH of the solution was then adjusted to pH 7–8 with 1 M Na_2_CO_3_ (p.a., Chempur, Poland, for model solutions) or 30% NaOH (p.a., Chempur, Poland, for real solutions). The molar ratio of the PGM precursor to the stabilizing agent and the reducer for the model solutions (PGM:PVP:reducer) was 1:1:1 and that for the real solution was 1:2:1.

### 3.3. Catalytic Reaction

To conduct the reactions of IB degradation photocatalysis, a reactor equipped with a UV–Vis lamp emitting light with a wavelength of 238–579 nm and a cooling system was applied. Exemplary, 150 mg of PGM–NPs@TiO_2_ and 150 cm^3^ of a 10 mg/dm^3^ IB solution was placed in the reactor, and stirred for specific time, for example 180 min. During the reaction, 1.5 cm^3^ samples were collected at 5, 10, 15, 30, 60, and 120 min from the initiation of the reaction. The samples were then centrifuged for 3 min at 15,000 rpm, and spectra were examined using a UV–Vis spectrometer in the range from 200 to 800 nm. Some tests were conducted without using the UV–Vis lamp for 7 days (168 h) to investigate, if the nanocatalysts were able to degrade IB without light contribution.

### 3.4. Apparatus

The concentrations of metal ions in the solutions before and after precipitation were measured using atomic absorption spectrometry (AAS ContrAA 300, Analytik Jena, Jena, Germany) at the following wavelengths: 266.0, 244.8, 343.5, 349.9, 248.3, 285.2, 213.9 and 324.7 nm for Pt(IV), Pd(II), Rh(III), Ru(III), Fe ions, Mg(II), Zn(II) and Cu(II), respectively.

The IB concentration was analyzed using a UV–Vis spectrometer (Specord 40, Analytik Jena, Jena, Germany) at λ_max_ = 222 nm against a standard curve.

The composition of the solutions after the degradation reaction and the concentration of IB in these solutions was determined to analyze the degradation paths using the chromatographic system UltiMate 3000 RSLC (Dionex, Thermo, Waltham, MA, USA) coupled to an API 4000 QTRAP triple quadrupole mass spectrometer with electrospray ionization (ESI) (from AB Sciex, Foster City, CA, USA) in negative ionization mode (UHPLC–MS/MS). For chromatographic separation, the Hypersil Gold C18 RP (100 mm × 2.1 mm × 1.9 µm) column by Thermo Scientific was used. The mobile phase consisted of a 5 mM solution of ammonium acetate (A) and methanol (B). The gradient program was: 50% B at 0 min, increased to 67% B in 3 min and held for 2 min, stepped to 100% in 3 min and held for 5.0 min; and the flow rate was 0.2 mL min^−1^. A post run time was set at 4.0 min for column equilibration before the next injection. The analytes were washed from the column using gradient elution. The separation was conducted at 35 °C, with a sample volume of 5 µL. The operating conditions for mass spectrometry for ibuprofen were as follows: curtain gas 20 psi, nebuliser gas, and auxiliary gas 50 psi, source temperature 400 °C, ion spray voltage -4500 V, and collision gas set to medium. Quantitative analysis of the compounds was performed in multiple reaction monitoring (MRM) mode. For the analytes, one transition of a deprotonated molecular ion and their respective ion products were chosen. These transitions (*m*/*z*) with associated declustering potentials (V) and collision energies (V) were as follows: ibuprofen 205 → 161, −50, −12. Degradation products of IB were determined by recording the total ion current chromatograms in the mass range *m*/*z* from 50 to 600 Da in both positive and negative ionization modes.

## 4. Conclusions

The PGM nanocatalysts on a TiO_2_ support facilitate the degradation reaction of IB. The exposure of the reaction medium to UV–Vis light further enhances this degradation process. The inclusion of PGMs in the catalyst shows a slight effect on the conversion degree of IB compared to reactions involving TiO_2_ without PGMs. Thus, the photocatalytic properties of TiO_2_ seem to play a crucial role in this process, while supported by the catalytic activity of PGMs.

UV–Vis spectroscopy reveals that Pt or Pd@TiO_2_ catalysts significantly enhance IB conversion, achieving approximately 80%, a notable increase of at least 20 percentage points compared to reactions conducted without a catalyst or with TiO_2_ alone, which reaches approximately 60% after 120 min. Interestingly, the differences in IB conversion values among all studied catalysts after 120 min of degradation, as determined by LC–MS/MS, remain slight (90–95%), irrespective of the catalyst’s presence or type.

As the PGMs@TiO_2_ nanocatalysts show promise for catalyzing the degradation of IB in aqueous solutions, potentially leading to improved water treatment processes, further studies on the degradation of other pharmaceuticals should be conducted. The challenges that should be addressed are investigating the stability of PGMs@TiO_2_ nanocatalysts under different conditions, to ensure their long-term effectiveness in water treatment applications and developing methods for the recovery and potential reuse of PGMs@TiO_2_ nanocatalysts to reduce costs and environmental impact.

## Figures and Tables

**Table 1:** pH before IB degradation without UV–Vis lamp and after 168 h (7 days) of the reaction course.

IB Degradation	Reaction pH		
	Without Catalyst	With TiO_2_ Alone	With Pd@TiO_2_
Before reaction	4.7	4.7	4.7
After 168 h	4.9	5.2	5.7

**Table 2:** The degree of IB conversion based on UV–Vis spectra obtained during the reaction enhanced by UV–Vis light.

Reaction Time, min	IB Conversion, %				
	Without Catalyst	TiO_2_	Pd@TiO_2_	Pt@TiO_2_	Pt/Fe/Pd@TiO_2_
UV-Vis spectroscopy					
5	22	34	35	70	No data.
15	38	46	50	74	
30	47	50	65	78	
60	50	63	76	80	
120	62	68	83	85	
LC–MS/MS technique					
5	40	40	52	50	88
15	52	69	53	65	91
30	87	77	63	82	92
60	88	84	70	91	92
120	93	90	91	95	94

**Table 3:** The k constants at the ambient temperature (based on the LC–MS/MS results).

Reaction Time, min	k, min^−1^				
	Without a Catalyst	TiO_2_	Pd@TiO_2_	Pt@TiO_2_	Pt/Fe/Pd@TiO_2_
5	0.1015	0.1048	0.1486	0.1367	0.4140
15	0.0495	0.0779	0.0509	0.0682	0.1625
30	0.0690	0.0500	0.0332	0.0581	0.0852
mean k	0.0733	0.0776	0.0776	0.0877	0.2205

**Table 4:** The degree of IB conversion obtained by other researchers in the presence of various NPs.

Type of NP	Conditions of Degradation Reaction	IB Conversion	Ref.
Magnetic Pd@Fe_3_O_4_	Enhanced by ultrasound and electrochemical reaction, 2 mg/dm^3^ IB, pH 5.2	100% in 60 min	[16]
Ag/Ag_2_O nanocomposite	Photoirradiation, 8 mg/dm^3^, room temperature, pH 3, 7, 9	30%	[22]
		15%	
		17%	
TiO_2_	Photocatalysis 222 nm, 0.03 g TiO_2_, pH 5.0	100% in 5 min	[23]
Co-P 800/PMS	10 mg/dm^3^ IB, 2 mM PMS, 0.1 g/dm^3^ catalyst, <23 °C, pH 7.6	90% in 120 min	[24]
BiOBr	Photocatalytic degradation—simulated solar light irradiation, 20 mg/dm^3^ IB, 0.5 g/dm^3^ BiOBr	100% in 20 min	[25]
MgFe_2_O_4_	Fenton-catalyst, 0.5 g/dm^3^ catalyst, 20 mmol/dm^3^ H_2_O_2,_ 10 mg/dm^3^ IB, pH 6.0	100% in 40 min	[33]
Ag/Bi–BiOBr	Photocatalytic degradation—simulated solar light irradiation, 20 mg/dm^3^ IB, 30 mg/100 cm^3^ Ag/Bi–BiOBr	92.3% in 60 min	[37]
MnCo_2_O_4_@FCNTs	Catalytic oxidation, 25.0 ± 0.2 °C, 10 mg/dm^3^ IB, pH 3–9	>90% in 10 min	[38]
ZnO	UV light irradiation 254 nm, 25 °C, 20 mg/dm^3^ IB	60% in 60 min	[39]
Biogenic Pt–NPs, Pd–NPs	Anaerobic conditions, 1 mg of the metallic cell NPs (25% of total mass composed by metal), 1 mg/dm^3^ IB, pH 5.3	0%	[40]

FCNT—functionalized multi-walled carbon nanotubes, Co–P 800—Co-doped carbon matrix, PMS—peroxymonosulphate activator.

## Data Availability

Data is contained within the article.

## Appendix A

The process for formation of nanocatalysts elaborated by our team to produce PGM-NPs from SACs is shown in the scheme.
